# Enablers and Barriers to HIV Services for Gay and Bisexual Men in the COVID-19 Era: Fusing Data Sets from Two Global Online Surveys Via File Concatenation With Adjusted Weights

**DOI:** 10.2196/33538

**Published:** 2022-06-27

**Authors:** George Ayala, Sonya Arreola, Sean Howell, Thomas J Hoffmann, Glenn-Milo Santos

**Affiliations:** 1 Alameda County Public Health Department San Leandro, CA United States; 2 Arreola Research San Francisco, CA United States; 3 Hornet San Francisco, CA United States; 4 Department of Epidemiology and Biostatistics University of California San Francisco San Francisco, CA United States

**Keywords:** COVID-19, HIV services, gay and bisexual men, sexual health

## Abstract

**Background:**

Gay and bisexual men are 26 times more likely to acquire HIV than other adult men and represent nearly 1 in 4 new HIV infections worldwide. There is concern that the COVID-19 pandemic may be complicating efforts to prevent new HIV infections, reduce AIDS-related deaths, and expand access to HIV services. The impact of the COVID-19 pandemic on gay and bisexual men’s ability to access services is not fully understood.

**Objective:**

The aim of this study was to understand access to HIV services at the start of the COVID-19 pandemic.

**Methods:**

Our study used data collected from two independent global online surveys conducted with convenience samples of gay and bisexual men. Both data sets had common demographic measurements; however, only the COVID-19 Disparities Survey (n=13,562) collected the outcomes of interest (HIV services access at the height of the first COVID-19 wave) and only the Global Men’s Health and Rights Survey 4 (GMHR-4; n=6188) gathered pre-COVID-19 pandemic exposures/covariates of interest (social/structural enablers of and barriers to HIV services access). We used data fusion methods to combine these data sets utilizing overlapping demographic variables and assessed relationships between exposures and outcomes. We hypothesized that engagement with the gay community and comfort with one’s health care provider would be positively associated with HIV services access and negatively associated with poorer mental health and economic instability as the COVID-19 outbreaks took hold. Conversely, we hypothesized that sexual stigma and experiences of discrimination by a health care provider would be negatively associated with HIV services access and positively associated with poorer mental health and economic instability.

**Results:**

With 19,643 observations after combining data sets, our study confirmed hypothesized associations between enablers of and barriers to HIV prevention, care, and treatment. For example, community engagement was positively associated with access to an HIV provider (regression coefficient=0.81, 95% CI 0.75 to 0.86; *P*<.001), while sexual stigma was negatively associated with access to HIV treatment (coefficient=–1.39, 95% CI –1.42 to –1.36; *P*<.001).

**Conclusions:**

HIV services access for gay and bisexual men remained obstructed and perhaps became worse during the first wave of the COVID-19 pandemic. Community-led research that utilizes novel methodological approaches can be helpful in times of crisis to inform urgently needed tailored responses that can be delivered in real time. More research is needed to understand the full impact COVID-19 is having on gay and bisexual men worldwide.

## Introduction

Gay men and other men who have sex with men (MSM; hereafter referred to as gay and bisexual men) [1] are 26 times more likely to acquire HIV than other adult men, and in 2019 represented nearly 1 in 4 new HIV infections worldwide [2,3]. While biological and social factors converge to elevate the risk for HIV acquisition and transmission [4], structural barriers such as sexual stigma, discrimination, and criminalization of sex between men impede access to and utilization of HIV testing, prevention, and treatment services [5,6]. Conversely, factors such as community engagement and having supportive health care providers enable service access and utilization for gay and bisexual men [7].

The world remains off track in meeting global HIV targets, especially for socially marginalized and criminalized groups. For example, surveys from 114 nationally representative data sets in 38 African countries with nearly 1.5 million sexually active adults aged 15-49 years conducted from 2003 to 2018 were examined to estimate trends in annual HIV testing and condom use during the last occurrence of highest-risk sex. These data were used to calculate the probability of reaching key Joint United Nations Programme on HIV/AIDS (UNAIDS) targets. Investigators observed limited progress and little chance of reaching global targets [8]. There is now concern that the COVID-19 pandemic may further complicate efforts to bend the HIV incidence curve; reduce AIDS-related deaths; and expand prevention, care, and treatment coverage [2]. Recent research suggests that COVID-19 is exacerbating challenges gay and bisexual men face in their attempts to access HIV and other sexual health services. A recent study found deleterious economic and mental health consequences of COVID-19 and public health responses among a global sample of gay and bisexual men [9]. The same study also found significant interruptions to HIV testing, prevention, treatment, and care services. The role of COVID-19–related social and structural factors in gay and bisexual men’s access to HIV-related services is less understood.

More evidence is needed for providing early and potentially critical programmatic and policy-related interventions in the era of COVID-19. This study utilized a statistical matching method that combined data sets (ie, data fusion) from two separate global online cross-sectional surveys to enable drawing inferences about the impact of the COVID-19 pandemic on gay and bisexual men’s ability to access services. Neither data set could address the question individually, as one had only outcomes and demographics and the other only exposures and demographics. Data fusion allowed us to relate outcomes to exposures across the data sets. Specifically, this approach allowed us to explore social and structural enablers of and barriers to HIV service access among gay and bisexual men worldwide during the early days of the COVID-19 pandemic. We hypothesized that engagement with the gay community and comfort with one’s health care provider would be positively associated with HIV services access and negatively associated with poorer mental health and economic instability despite the challenges brought about by COVID-19 outbreaks. Conversely, we hypothesized that sexual stigma and experiences of discrimination by a health care provider would be negatively associated with HIV services access and positively associated with poorer mental health and economic instability.

Our hypotheses are informed by social ecological theory, which suggests that various factors at structural, community, interpersonal, and individual levels facilitate or impede access to resources such as HIV and other health services. Social ecological theory is useful for identifying high-impact leverage points in the successful implementation of health-promoting interventions and for strategic alignment of policy and services across a continuum of population health needs [10,11].

## Methods

### Study Design

Our study used data collected from two independent surveys conducted with gay and bisexual men. The first, Global Men’s Health and Rights Survey 4 (GMHR-4), was designed to explore correlates to HIV services access and utilization. GMHR-4 was launched on September 4, 2019, and closed on March 31, 2020 [12,13]. Slightly over 1% of participants took the survey after February 2020. Earlier versions of the survey are described elsewhere in greater detail [5,14]. GMHR-4 data were collected from a nonprobability internet sample of gay and bisexual men, recruited via organizational outreach, email listservs, gay dating apps, and websites. Participants were invited to complete a 20- to 30-minute online survey. Eligible participants who consented to take the survey needed to identify as male (cisgender or transgender); have had sex with another man in the last 6 months; be 18 years or older; and able to complete the online survey in Arabic, Chinese, English, French, Indonesian, Portuguese, Russian, Spanish, Swahili, or Vietnamese. No geographical restrictions were applied.

The second survey, COVID-19 Disparities Survey, was administered by Hornet between April 16, 2020, through May 4, 2020 [9]. Hornet is a free, smartphone-based gay social networking app with over 25 million users worldwide. Its users are predominantly gay and bisexual men. The COVID-19 Disparities Survey was a brief, 10- to 15-minute questionnaire, which sought to explore the impacts of COVID-19 on economic status, mental health, and HIV services access among Hornet users. Eligible participants were Hornet users, 18 years or older.

### Ethics Approval

Ethical approval for the use of GMHR-4 data was obtained from the Western Institutional Review Board, which determined that GMHR-4 was exempt under 45 CFR 46.104(d)(2) (#1-1174358-1). Study procedures for the COVID-19 Disparities Survey were reviewed by the Johns Hopkins School of Public Health Institutional Review Board, which designated the protocol as exempt under Category 4.

### Measures

Both surveys included *demographic questions* (eg, age, country of residence, sexual orientation, gender identity, relationship status, racial/ethnic minority status, ability to meet one’s basic financial needs, health care coverage, and HIV status). GMHR-4 included questions responded to on 5-point Likert scales, which asked about: (1) *engagement with the gay community,* assessed on a 10-item scale (Cronbach α=.72), including “During the past 6 months, how often have you participated in gay/bisexual/MSM with social groups?” with a response scale ranging from 1=never to 5=more than 12 times*;* (2) *comfort with one’s health care provider,* assessed on a 3-item scale (Cronbach α=.85), including “In your country, how comfortable do you feel discussing your sexual health concerns with your health care provider?” where 1=very uncomfortable and 5=very comfortable; (3) experiences of *sexual stigma* (ie, homophobia) using a 7-item scale (Cronbach α=.82), including “In your country, how many people believe that male homosexuals are disgusting?”, where 1=none, 2=a few, 3=some, 4=most, 5=all; and (4) *provider discrimination*, a 6-item scale (Cronbach α=.87), including “In the last 6 months, has a health care provider refused to treat you because you are gay/bisexual/MSM?” where responses ranged from 1=no-never to 5=yes more than 5 times.

The COVID-19 Disparities Survey asked participants about the impact COVID-19 was having on their economic situation; mental health; and ability to access HIV testing, prevention, care, and treatment services. *Economic impact* was assessed using the question: “How much are you expecting your income to reduce because of the COVID-19 crisis?” The economic impact question used a categorical response scale from 0% to 100%, ordered in 10-point increments. *Mental health* was assessed using validated items from the Patient Health Questionnaire-4 (PHQ-4), which is used to screen for depression and anxiety; scores ≥3 indicate psychological distress [15].

**Figure 1:**
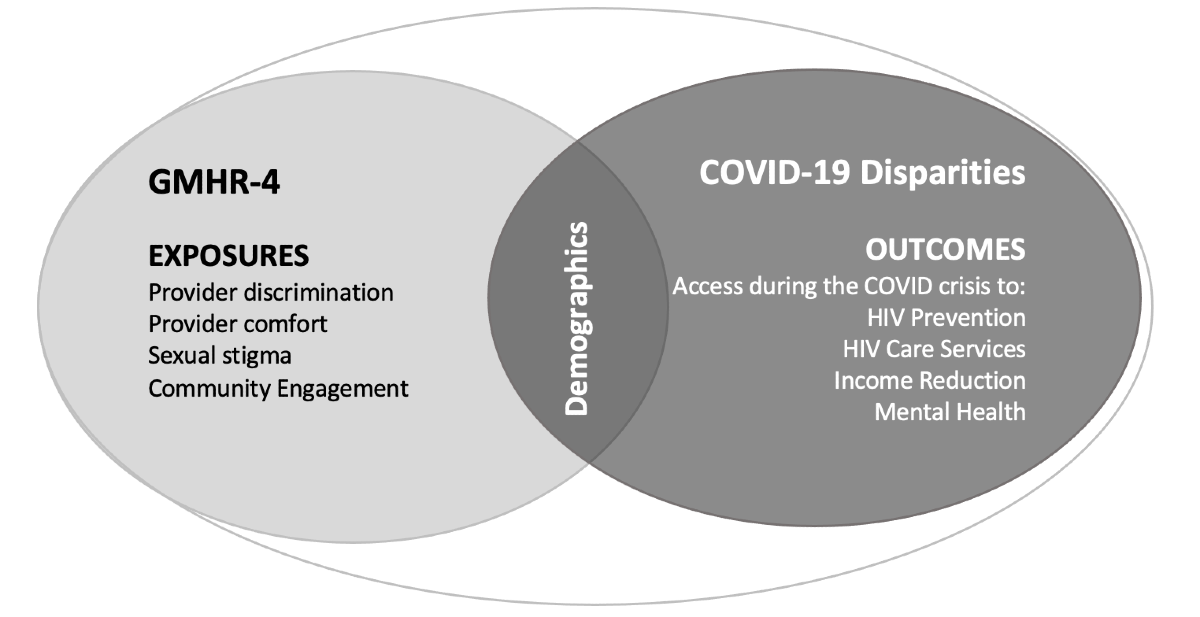
Venn diagram of Global Men’s Health and Rights Survey 4 (GMHR-4) and COVID-19 Disparities survey measures used for data fusion analysis. Demographic measures that overlap between the two studies' samples include: age, country of residence, sexual orientation, gender identity, relationship status, racial/ethnic minority status, ability to meet one’s basic financial needs, health care coverage, and HIV status.

HIV services impact measures asked whether participants experienced changes in *access to condoms, HIV onsite HIV testing,* and *pre-exposure prophylaxis* (PrEP), such as “Do you feel you have access to HIV prevention strategies during the COVID-19 crisis, i.e., condoms, PrEP, onsite HIV testing?” The 5-point Likert scale response options ranged from “definitely yes” to “definitely no.” For participants living with HIV, the survey asked about *access to HIV providers,* such as “Since the beginning of the COVID-19 crisis, have you been able to see your HIV provider if you needed to?”, with the following response options: “Yes, in person”; “Yes, via telemedicine”; “No, because of reduced hours”; “No, because it is closed”; “Not applicable.” In addition, the survey assessed *treatment access* by asking, “Do special measures related to COVID-19 impact your ability to access or refill your HIV medicine?” Response options included: “I cannot access or refill my HIV medicine”; “I can access or refill my HIV medicine, but access is burdensome or complicated”; and “Not applicable.” Measures used for the data fusion analysis (described below) are depicted in Figure 1.

### Statistical Analysis

Because we were examining data from two different surveys with only partial variable overlap (demographics in both data sets, exposures in one data set, and outcomes in the other data set), and we were interested in associations between nonoverlapping variables across these questionnaires, we utilized statistical matching (ie, data fusion) methods to combine the data sets [16,17] in STATA v15 (College Station, TX) with the community-contributed *smpc* and *smmatch* programs [18]. The data fusion method works as follows: data set A (which contains variables Y and X) and data set B (which contains variables X and Z) are concatenated and weighted. Y is regressed on X and Z is regressed on X, and a prespecified partial correlation *ρ*_Y,Z|X_ (discussed below) is used to calculate joint regression coefficients and predict Y and Z values in the concatenated data set. Each individual missing Z (ie, from data set A) is matched to the closest new predicted Z in data set B. After matching, the observed value of the match is imputed as the missing value. A similar process is used for individuals missing Y (ie, from dataset B) [19]. In practice, a range of 5 partial correlation values has been shown to work well and reduce bias [17], and results can then be combined utilizing existing equations and frameworks from multiple imputation (but we note that the method is different than the usual multiple imputation). All predictors and outcomes are treated as continuous variables.

To make an initial informed estimate of the partial correlations between the outcomes from the COVID-19 Disparities Survey data set and the GMHR-4 predictors in the imputation prediction regression models, we calculated the partial correlations between each of the GMHR-4 predictors with the measure that we believed was the closest proxy to each of the outcomes in the COVID-19 data set, while accounting for the jointly observed demographic characteristics (income, education, relationship status, urbanicity [urban vs rural], racial/ethnic minority status, health insurance, and region [Global North vs Global South]). For example, we calculated the partial correlation between the exposure, community engagement, and access to HIV testing in the GMHR-4 data set, while accounting for the jointly observed demographic variables. We then used this partial correlation value in the prediction model for community engagement and the outcome of access to HIV testing during COVID-19 in the COVID-19 Disparities Survey data set. That is, we assumed that the partial correlation between community engagement and access to HIV testing in GMHR-4 was a reasonable approximation for the partial correlation between community engagement in GMHR-4 and access to HIV testing during COVID-19 in the COVID-19 Disparities Survey data set. The range of partial correlations used in the fusion procedure included this initial informed estimate, ±5%, and ±10%.

For each partial correlation value and resulting fused data set, we then performed linear regressions between the imputed outcomes and exposures [17]. In these models, outcomes included access (during the COVID-19 crisis) to onsite HIV testing, condoms, PrEP, HIV care, HIV treatment, and mental health services; exposures (covariates of interest) included sexual stigma, provider discrimination, provider comfort, and engagement in the gay community. The models also adjusted for income, education, relationship status, urbanicity (urban vs rural), racial/ethnic minority status, health insurance, and region (Global North vs Global South). In sensitivity analyses, we limited the covariates in the regression models to the demographic characteristics above, exclusive of urbanicity and region.

## Results

### Sample Characteristics/Matched Variables

A total of 19,643 observations from gay and bisexual men were included in this study after combining outcomes from the GMHR-4 (n=6189) with exposures from the COVID-19 Disparities Survey (n=13,454) through overlapping demographics via data fusion (see Figure 1). Among the total sample, 44.00% (8643/19,643) of participants were under the age of 30 years and 57.00% (11,197/19,643) indicated an inability to financially meet their basic needs. Participants had a high level of education, with 54.00% (10,607/19,643) having completed college. Global northerners (participants from Europe, Canada, the United States, Australia, and New Zealand) and southerners (participants from Africa, Asia, the Caribbean, Latin America, the Middle East, and Pacific Islands) were nearly equally represented in the combined data set. Higher proportions of GMHR-4 study participants reported an HIV-positive status, were from the Global South, and had completed a college education when compared with study respondents from the COVID-19 Disparities Survey. Participant demographics are more fully summarized in Table 1.

**Table 1:** Participant demographic characteristics jointly observed across both data sets.

Characteristics		Total^a^ (N=19,643), n (%)	GMHR-4^b^ (n=6189), n (%)	COVID-19 Disparities (n=13,454), n (%)
**Age (years)**				
	<20	1287 (6.55)	537 (8.68)	750 (5.57)
	20-29	7234 (36.83)	2687 (43.42)	4547 (33.80)
	30-49	9240 (47.04)	2528 (40.85)	6712 (49.89)
	50+	1878 (9.56)	433 (7.00)	1445 (10.74)
**Economic status**				
	Not able to meet needs well	11,275 (57.40)	3982 (64.34)	7293 (54.21)
	Able to meet needs well	8368 (42.60)	2207 (35.66)	6161 (45.79)
**Education**				
	Did not complete college	9031 (45.98)	1553 (25.09)	7478 (55.58)
	Completed college	10,612 (54.02)	4636 (74.91)	5976 (44.42)
**Relationship status**				
	In a relationship	5725 (29.15)	1673 (27.03)	4052 (30.12)
	Not in a relationship	13,918 (70.85)	4516 (72.97)	9402 (69.88)
**Location of residence**				
	Not in a city/urban area	4070 (20.72)	831 (13.43)	3239 (24.07)
	Resides in a city/urban area	15,573 (79.28)	5358 (86.57)	10,215 (75.93)
**Racial and ethnic background**				
	Not a racial or ethnic minority	16,117 (82.05)	4733 (76.47)	11,384 (84.61)
	Racial or ethnic minority	3526 (17.95)	1456 (23.53)	2070 (15.39)
**Health insurance**				
	No	5398 (27.48)	1908 (30.82)	3490 (25.94)
	Yes	14,245 (72.52)	4281 (69.17)	9964 (74.06)
**Region**				
	Global South	9671 (49.23)	5563 (89.89)	4108 (30.53)
	Global North	9741 (49.59)	598 (9.66)	9143 (67.96)
**HIV status**				
	Not living with HIV	17,194 (87.53)	5173 (83.58)	12,021 (89.35)
	Living with HIV	2449 (12.47)	1016 (16.42)	1433 (10.65)

^a^Values may not necessarily add to column totals due to missing responses from participants.

^b^GMHR-4: Global Men’s Health and Rights Survey 4.

### Partial Correlations

Partial correlations between each GMHR-4 predictor with the measure that we believed represented the closest proxy to each of the outcomes in the COVID-19 data set were calculated and are presented in Table 2. Partial correlations were estimated while adjusting for jointly observed demographic characteristics, including age, country of residence, sexual orientation, gender identity, relationship status, racial/ethnic minority status, ability to meet one’s basic financial needs, health care coverage, and HIV status.

**Table 2:** Partial correlations between Global Men’s Health and Rights Survey 4 (GMHR-4) predictor and GMHR-4 proxy measures for COVID-19 outcome variables^a^.

Predictors	HIV testing	Condoms	PrEP^b^	Access to HIV provider	Access to ART^c^ refills	Low income	Poor mental health
Community engagement	0.08	0.05	0.13	0.11	0.11	0.02	–0.06
Comfort with provider	0.21	0.19	0.25	0.26	0.26	–0.17	–0.15
Sexual stigma	–0.15	–0.19	–0.21	–0.21	–0.18	0.17	0.15
Provider discrimination	–0.07	–0.08	–0.06	–0.05	–0.04	0.09	0.10

^a^Values calculated for partial correlations were used as anchors for the range of partial correlations used in smpc and smmatch, with the range set at ±5% and ±10% of values.

^b^PrEP: pre-exposure prophylaxis.

^c^ART: antiretroviral therapy.

### Enablers and Barriers to HIV Services

Although sexual stigma was commonly reported by study participants (mean 3.53, SD 0.61), discrimination from one’s health care provider was low (mean 1.14, SD 0.39). Comfort with one’s provider was also frequently reported (mean 2.98, SD 1.25). Our study found poor community engagement, as evidenced by a low mean score (mean 1.32, SD 0.43).

### Access to HIV Prevention

Study participants reported relatively high access to HIV testing (mean 3.7, SD 0.43) and condoms (mean 4.6, SD 0.9), but suboptimal access to PrEP (mean 3.2, SD 1.4). Our study confirmed hypothesized associations between enablers of and barriers to HIV prevention. Community engagement and comfort with one’s health care provider were positively associated with access to HIV testing, condoms, and PrEP. Conversely, sexual stigma and experiences of provider discrimination were negatively associated with access to the same set of prevention services. Associations were statistically significant (*P*<.005). Coefficients and confidence intervals are shown in Table 3.

**Table 3:** Associations between hypothesized predictors and access to HIV prevention^a^.

Predictors	HIV onsite testing access			Condom access				PrEP^b^ access		
	Coef^c^	95% CI	*P* value	Coef	95% CI	*P* value	Coef		95% CI	*P* value
Community engagement	1.16	1.14 to 1.18	<.001	0.95	0.92 to 0.99	<.001	1.23		1.16 to 1.30	<.001
Comfort with provider	0.78	0.77 to 0.79	<.001	0.69	0.68 to 0.70	<.001	0.90		0.89 to 0.91	<.001
Sexual stigma	–0.86	–0.87 to-0.84	<.001	–1.05	–1.08 to –1.02	<.001	–1.05		–1.07 to –1.03	<.001
Provider discrimination	–0.96	–1.01 to –0.92	<.001	–0.81	–0.84 to –0.77	<.001	–1.17		–1.19 to –1.14	<.001

^a^Regression models also adjusted for income, education, relationship status, urbanicity (urban vs rural), racial/ethnic minority status, health insurance, and region (Global North vs Global South) as covariates. In sensitivity analyses omitting urbanicity and region, results were similar with respect to magnitude and level of significance of estimates.

^b^PrEP: pre-exposure prophylaxis.

^c^Coeff: regression coefficient.

### Access to HIV Care and Treatment

Our study found access to HIV care (mean 2.4, SD 0.8) and HIV treatment (mean 2.1, SD 1.2) to be low. Enablers of and barriers to HIV care and treatment were significantly associated in the predicted directions. For example, community engagement was positively associated with access to an HIV provider and sexual stigma was negatively associated with access to HIV treatment. A detailed summary of associations is presented in Table 4.

**Table 4:** Associations between hypothesized predictors and access to HIV care and treatment^a^.

Predictors	HIV provider access			Access to ART^b^ refills		
	Coef^c^	95% CI	*P* value	Coef	95% CI	*P* value
Community engagement	0.81	0.75 to 0.86	<.001	1.23	1.19 to 1.27	<.001
Comfort with provider	0.54	0.53 to 0.55	<.001	0.79	0.78 to 0.80	<.001
Sexual stigma	–0.87	–0.95 to –0.78	<.001	–1.08	–1.11 to –1.05	<.001
Provider discrimination	–0.77	–0.81 to –0.73	<.001	–1.13	–1.17 to –1.09	<.001

^a^Regression models also adjusted for income, education, relationship status, urbanicity (urban vs rural), racial/ethnic minority status, health insurance, and region (Global North vs Global South) as covariates. In sensitivity analyses omitting urbanicity and region, results were similar with respect to magnitude and level of significance of estimates.

^b^ART: antiretroviral therapy.

^c^Coef: regression coefficient.

### Mental Health

The mean PHQ-4 score was 4.7, indicative of prevalent depression and anxiety among respondents who were included in this study. Community engagement and comfort with one’s health care provider were each negatively associated with poorer mental health. However, poorer mental health outcomes were significantly associated with experiences of sexual stigma and provider discrimination (see Table 5).

**Table 5:** Associations between hypothesized predictors and poorer mental health^a^.

Predictors	Poorer mental health (PHQ-4^b^)		
	Coef^c^	95% CI	*P* value
Community engagement	–3.03	–3.19 to –2.87	<.001
Comfort with provider	–2.19	–2.23 to –2.15	<.001
Sexual stigma	2.48	2.42 to 2.54	<.001
Provider discrimination	3.15	2.94 to 3.35	<.001

^a^Regression models also adjusted for income, education, relationship status, urbanicity (urban vs rural), racial/ethnic minority status, health insurance, and region (Global North vs Global South) as covariates. In sensitivity analyses omitting urbanicity and region, results were similar with respect to magnitude and level of significance of estimates.

^b^PHQ-4: Patient Health Questionnaire-4.

^c^Coef: regression coefficient.

### Economic Impact

Although the mean score for the question assessing anticipated income reduction was low (2.4), the SD (3.6) suggests broad variability in participant responses. As displayed in Table 6, regression analyses confirmed hypothesized associations between predictor and outcome variables of interest, with one important exception: community engagement was positively associated with anticipated income reduction.

**Table 6:** Associations between hypothesized predictors and economic instability^a^.

Predictors	Anticipated income reduction during COVID-19		
	Coef^b^	95% CI	*P* value
Community engagement	0.84	0.74 to 0.93	<.001
Comfort with provider	–0.65	–0.65 to –0.64	<.001
Sexual stigma	0.74	0.73 to 0.75	<.001
Provider discrimination	0.89	0.85 to 0.92	<.001

^a^Regression models also adjusted for income, education, relationship status, urbanicity (urban vs rural), racial/ethnic minority status, health insurance, and region (Global North vs Global South) as covariates. In sensitivity analyses omitting urbanicity and region, results were similar with respect to magnitude and level of significance of estimates.

^b^Coef: regression coefficient.

## Discussion

### Principal Findings

To our knowledge, this is the first community-led, HIV-related research study to systematically combine data sets via data fusion from two separate online surveys of gay and bisexual men. The strategy enabled us to compare variables that would otherwise not be comparable. Specifically, we could combine outcomes from one data set with exposures from the other data set with a fusion process through the overlap [18]. This approach allowed us to confirm hypothesized associations between sexual stigma, provider discrimination, community engagement, and comfort with one’s health care provider, each experienced prior to the global onset of the COVID-19 pandemic, with access to HIV services, income reduction, and mental health impact at the height of the pandemic’s first surge.

Our study suggests that experiences of sexual stigma and provider discrimination continue to be common and likely persist through the COVID-19 pandemic. In addition, despite low overall levels of engagement with the gay community, when reported, community engagement may be moderating the deleterious effects of sexual stigma and provider discrimination on mental health and economic security. This may be because communities are sources of information, safety, support, and affinity [20-24]. Although community engagement was positively associated with anticipated reductions in income, this finding makes sense if study participants are actively engaging community-based or -led organizations for support. Study findings confirm prior research showing the enabling effects of community engagement and comfort with one’s health care provider on access to HIV prevention, testing, treatment, and care services for gay and bisexual men [25]. Moreover, HIV and other health services are more likely to be perceived as accessible and to be utilized if they are delivered by peers [26-28].

All exposures or predictors were assessed using scales contained in GMHR-4, based on data collected in the weeks and months prior to the pandemic. All outcomes were measured using items from the COVID-19 Disparities Survey. Although we cannot directly infer causal relationships between predictor and outcome variables, measures utilized asked COVID-19 Disparities Survey participants to consider COVID-19 in their responses, allowing us the unique opportunity to infer associations beyond the time parameters prescribed by GMHR-4. Our findings point to actionable factors that both enable and inhibit access to HIV services for gay and bisexual men in the COVID-19 era.

### Strengths and Limitations

There are some study limitations that are important to mention. First, both the GMHR-4 and COVID-19 Disparities Survey utilized online convenience samples, recruited through networks of advocates, service providers, and online dating apps. The study is therefore subject to selection bias for gay and bisexual men who are connected through networks and for whom internet-based technologies are more easily available. Study participants may thus have greater access to information and motivation to respond to surveys. Based on the sociodemographic characteristics of the sample, participants may likely have been gay and bisexual men who were less affected by the negative consequences of the COVID-19 pandemic. Consequently, findings reported here may reflect an underestimation of the true magnitude of COVID-19’s impact. In addition, the COVID-19 Disparities Survey was conducted at different stages of the epidemic’s spread and the magnitude of national responses likely varied from country to country. Convenience sampling also violates the assumption of the data fusion method that the two data sets were drawn as simple random samples from the same population [16], which may also bias our results. In addition, we note that our two data sets had more pronounced differences from each other in education and region. However, the matching procedure itself used to impute values is based on matching to similar covariate values. Moreover, results depended on our specification and assumptions used in the partial correlation for imputation. Nevertheless, we used estimates in our partial correlation calculation that we expected to be close and further combined over a range of partial correlation values [17]. Additional research is needed to determine the accuracy of our assumptions in the partial correlations we calculated. Like other observational studies, there may be other unmeasured confounders (eg, mental health, socioeconomic status) that may be associated with our exposures of interest and access to HIV services. Finally, the results relied on data that are cross-sectional in nature, which precludes our ability to examine temporal changes in predictors and outcomes measured. Further research, including qualitative studies, are needed to fully explore: (1) unequal access to HIV services, including their causes; (2) factors that enable access to both services and health; and (3) the impact COVID-19 is having on gay and bisexual men worldwide.

Despite these limitations, our study underscores the continued need to better understand and address impediments to service access among gay and bisexual men, especially in the context of the COVID-19 pandemic. Key strengths of this study include the range of domains used that can be harnessed for future research related to the HIV and COVID-19 pandemics and their impact on vulnerable populations. These include individual financial security, mental and sexual health, access to services, and program utilization. Moreover, the data sets used include samples from countries hardest hit by COVID-19, including Brazil, France, Mexico, and Russia.

Studies highlighting factors thought to enable access to HIV services are rare among gay and bisexual men [29]. Further, barriers to HIV services unique to international samples of gay and bisexual men are only sporadically studied [5,6,30,31], and are not universally and specifically addressed in national HIV responses [32]. Understanding the enablers of and barriers to HIV services access is critically important to getting the world back on track to achieving zero new infections, zero AIDS deaths, and zero discrimination [33]. Having a full and nuanced grasp of service enablers and barriers is especially important now, as we witness the impact of a second, unrelated global pandemic. This is because pre-existing vulnerabilities may become exacerbated during times of crisis, moderated to the extent that enabling factors are consistently and strategically buttressed with funding and technical support [34].

### Conclusions

Community-led research employing novel methodological approaches are paramount during times of crisis. The use of approaches such as data fusion to combine data sets can help to quickly clarify salient enabling factors rapidly and cost-efficiently, as well as expose the economic, mental health, and service impacts of sexual stigma and provider discrimination. Such information can potentially lead to tailored responses delivered in real time, which can be critically important during public health emergencies such as that represented by the COVID-19 pandemic. Community-led, methodologically creative, and cost-efficient approaches should be encouraged and funded, especially during such times.

Our study specifically highlights the importance of reinforcing enablers such as community engagement and comfort with one’s health care provider, while addressing stigma and discrimination as critically and equally central to ensuring equitable HIV services access among gay and bisexual men worldwide. Although not new, the findings reported here suggest that addressing factors that enable and deter access to HIV services may be especially important in buffering against the mental health and economic impacts of new and unrelated pandemics. Moreover, our study raises the question of whether the COVID-19 pandemic has resulted in worsening HIV services access among gay and bisexual men, a question that remains open and ready for further research. Future research is needed, including prospective studies of gay and bisexual men that more deeply examine the associations between exposures and outcomes of interest within the same sample. Future studies should also examine the concerns of gay and bisexual men more comprehensively, beyond those related to HIV, in a world transfixed and transformed by COVID-19.

## Abbreviations

GMHR-4: Global Men’s Health and Rights Survey-4
MSM: men who have sex with men
PHQ-4: Patient Health Questionnaire-4
PrEP: pre-exposure prophylaxis
UNAIDS: Joint United Nations Programme on HIV/AIDS
